# Access to and Utilization of Endocrine Therapy Among Breast Cancer Survivors in Botswana

**DOI:** 10.1200/GO.24.00180

**Published:** 2024-12-12

**Authors:** James R. Wester, Rachel B. Wagner, Bosa Motladiile, Isaac Nkele, Nkhabe Chinyepi, Moeketsi J. Makhema, Tara M. Friebel-Klingner, Peter Vuylsteke, Shahin Lockman, Scott Dryden-Peterson, Racquel E. Kohler

**Affiliations:** ^1^ Department of Surgery, Hospital of the University of Pennsylvania, Philadelphia, PA; ^2^ Center for Cancer Health Equity, Rutgers Cancer Institute of New Jersey, New Brunswick, NJ; ^3^ Botswana Harvard Health Partnership, Princess Marina Hospital, Gaborone, Botswana; ^4^ Department of Surgery, University of Botswana, Gaborone, Botswana; ^5^ Rutgers Global Health Institute, New Brunswick, NJ; ^6^ Department of Internal Medicine, University of Botswana, Gaborone, Botswana; ^7^ Division of Infectious Diseases, Brigham and Women's Hospital, Boston, MA; ^8^ Department of Medicine, Harvard Medical School, Boston, MA

## Abstract

**PURPOSE:**

Breast cancer (BC) is the most common female cancer worldwide, and the burden is increasing across sub-Saharan Africa. For women with hormone receptor–positive (HR+) cancers, endocrine therapy (ET) taken for 5-10 years can reduce the risk of recurrence by half. We explored experiences with ET and barriers to utilization among survivors in Botswana.

**METHODS:**

We recruited women with nonmetastatic disease from a survivorship cohort who had undergone mastectomy within 1-5 years for semi-structured interviews to explore experiences with treatment. This thematic content analysis focused on ET, so the sample included women with HR+ cancer who should have received ET and HR− women who reported taking ET.

**RESULTS:**

We analyzed interviews with 19 women (mean age 54 years, 42% stage I/II, 58% stage III). Three key themes were identified: (1) limited provider counseling, (2) challenges refilling prescriptions at public pharmacies, and (3) high medication and transportation costs associated with private pharmacies. Subthemes included immunohistochemistry result communication, lack of knowledge, frequent public pharmacy stockouts, inconvenient prescription refill policies, and medication switching and discontinuation, especially among participants with low socioeconomic positions (SEPs). Women's persistence, SEP, and financial support facilitated refills. Although some experienced side effects, they were not a common reason for discontinuation.

**CONCLUSION:**

BC survivors in Botswana face multilevel barriers to accessing and adhering to ET. Provider and health system improvements are needed to effectively communicate ET importance and increase access to consistently available and affordable medication.

## Teaser

Breast cancer survivors in Botswana face structural and health system barriers to endocrine therapy.

## INTRODUCTION

Globally, breast cancer (BC) was the most commonly diagnosed malignancy and the leading cause of cancer death among females in 2022.^1^ The incidence is projected to continue increasing in multiple countries across sub-Saharan Africa (SSA).^2-4^ SSA has the highest incidence-to-mortality ratio, largely due to 50%-90% of cases being diagnosed with advanced disease and lack of screening.^5-7^ In many of these settings, BC disproportionately affects women under age 50 years,^8,9^ and nearly two thirds of patients in SSA are hormone receptor–positive (HR+).^10,11^

**CONTEXT**

**Key Objective**
What are breast cancer (BC) survivors' experiences and challenges with initiating, adhering to, and accessing endocrine therapy (ET) in Botswana?
**Knowledge Generated**
Overall, adherence was suboptimal due to individual financial, provider-level, and health system–level barriers. Despite experiencing side effects, survivors were motivated to adhere. Limited counseling on ET benefits and side effects, medication stockouts at public pharmacies, and high costs associated with private pharmacies contributed to medication switching and discontinuation.
**Relevance**
Given the burden of hormone receptor–positive BC in sub-Saharan Africa, prioritizing ET affordability and expanding access remain critical to improve BC care quality and outcomes in the region.

For women with HR+ BC, endocrine therapy (ET) for 5-10 years can reduce the risk of recurrence by half.^12-15^ Tamoxifen, which has been on the WHO Essential Medicines List for a few decades,^16^ is recommended as part of comprehensive BC management^17,18^ and an affordable aspect of survivorship care.^19-21^ However, studies from multiple low- and middle-income countries (LMICs) have found that adherence varies, particularly among younger women.^22-25^ Reasons for nonadherence in LMICs include side effects,^26-28^ costs,^29-31^ and socioeconomic position (SEP).^32-34^ Comorbidities,^35^ like living with HIV,^25^ also influence adherence. However, few studies have examined ET experiences and adherence in SSA.^36,37^

In Botswana, a middle-income country in SSA, the majority (68%) of patients with BC are diagnosed with advanced disease and nearly 70% have HR+ cancers.^38,39^ The median age at diagnosis is 54 years.^11^ However, women living with HIV (WLWH) tend to be diagnosed younger and have worse survival.^38^ Reasons for poor outcomes and barriers to guideline-concordant treatment include low awareness, cancer stigma, limited pathology resources, medication stockouts, and transportation costs.^40-43^ However, none of these studies explicitly focused on ET. Therefore, we conducted a qualitative study to explore BC survivors' ET experiences in Botswana to identify challenges and potential solutions to improving adherence and survivorship care.

## METHODS

### Setting

Botswana has a strong public health care system that provides free access to cancer care for citizens. Medications, including tamoxifen, are covered at public facilities; however, at private facilities, medication must be purchased by patients or through medical insurance.^44,45^ No formal breast screening program exists, although some nongovernmental organizations provide opportunistic screening.^46^ During the study, most diagnostic and therapeutic services (eg, chemotherapy, surgery) were provided at two public and two private hospitals in the two largest cities. One of the private hospitals was the primary facility offering radiotherapy to the entire country. Tumor board meetings are not mandatory. Diagnostic work-up, including immunohistochemistry (IHC) testing, is available through the National Health Laboratory.^47^ However, IHC results typically return weeks to months after primary diagnosis,^48^ meaning women may be prescribed ET before result availability. Approximately 85% of patients with BC receive modified radical mastectomy over breast-conserving lumpectomy.^39^

### Recruitment

This protocol was approved by the authors' Institutional Review Board (Pro#18653). We identified and recruited participants from the prospective Thabatse Cancer Cohort (TCC),^49-52^ a survivorship cohort established in 2010.^49,51,52^ Briefly, the cohort enrolls 500-600 patients entering cancer care annually (approximately 80% of the nationwide total) at oncology treatment facilities across Botswana. Upon enrollment, participants are interviewed, medical records are abstracted, and outcomes are followed for 5 years.

The present study is a subanalysis of a larger qualitative study on experiences after mastectomy. In the broader study, eligible participants were survivors of nonmetastatic BC, age >18 years, who underwent mastectomy 1-5 years from enrollment. Potential participants were identified from TCC and contacted via phone to schedule a time and location for the interview. Participants were approached purposively to ensure a variety of perspectives were represented. Specifically, we recruited to obtain differences concerning stage and age at diagnosis, geography, and time after mastectomy. Menopausal status was not available in TCC data. Verbal and written informed consent were obtained in Setswana before interviews began. We conducted interviews from February to April 2022. Although we recruited 23 participants overall, this analytic sample was restricted to 19 women who had documented HR+ IHC results or reported experiences with ET. The four participants who had pathologically confirmed HR− BC were excluded from this analysis.

### Data Collection

Clinical and demographic information was abstracted from TCC. The semi-structured interview guide covered women's understanding of, and experiences with, ET after mastectomy. We developed the guide based on expert consensus including input from coauthors, TCC staff, and hospital clinicians. We conducted two pilot interviews at the public referral hospital, before finalizing the guide. Example questions are in Table 1. The guide also covered mastectomy and quality of life; however, this analysis reports solely on ET.

**TABLE 1:** Interview Topics and Example Questions About Experiences With Endocrine Therapy

Topic	Example Questions
Initiation	Were you told ever to start hormonal estrogen therapy?
	How did you decide you wanted to start hormonal estrogen therapy?
	Did you receive any hormonal estrogen therapy (like tamoxifen or anastrozole)?
	What prevented you from starting it?
Adherence	Did you experience any side effects?
	Has there ever been a time when you did not take it?
	Why did you stop taking it?
Access	Have you had challenges getting the pills?
	Was there ever a time when you could not get pills from the pharmacy?

Interviews were conducted by a female medical trainee who is a native Setswana speaker (B.M.) with an English-speaking, male medical trainee (J.R.W.), who both received training in qualitative research methods and were advised by a social scientist with qualitative expertise (R.E.K.). Interviews in Setswana took place either in the participant's residence or in hospital examination rooms. Recordings were professionally transcribed directly into English and deidentified, coded, and thematically analyzed. Data collection for the broader study ended upon reaching saturation, when no new emerging themes occurred.^53^

### Data Analysis

Study team members (J.R.W., B.M., and R.E.K.) met to discuss and determine code application in *Atlas.ti 23*, which was largely based on interview guide topics and revised throughout the coding process. Two coders (J.R.W. and B.M.) independently applied codes; 68% of interviews were double-coded. Coders met to reconcile discrepancies and reach consensus on definitions and examples of specific codes and concepts. Following an immersion-crystallization approach, the analytic team (J.R.W., R.B.W., and R.E.K.) wrote memos analyzing and summarizing coding reports for thematic analysis. Findings were discussed with the study team with expertise in health services research, cancer prevention and control, primary care, and BC care in Botswana. We met routinely to reflect on, corroborate, and synthesize key findings,^54,55^ which were reported according to COREQ guidelines.^56^ This analysis focuses on ET experiences of women with HR+ cancer who should have received ET, and we briefly report on women with HR− cancer who described taking ET.

## RESULTS

**FIG 1:**
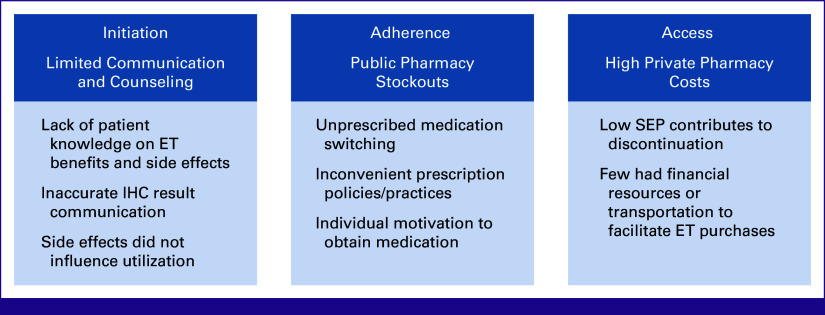
Major themes and subthemes influencing ET initiation, access, and adherence. ET, endocrine therapy; IHC, immunohistochemistry; SEP, socioeconomic position.

We identified factors across multiple ecological levels, which affected ET initiation, adherence, and access (Fig 1). Major themes at the provider and health system levels were limited counseling, frequent stockouts at public pharmacies, and high costs associated with private pharmacies. Additional thematic findings revealed that issues delivering accurate IHC results about HR status affected ET initiation and left many women confused about the purpose of medication. Challenges at public pharmacies including medication shortages and inconvenient prescription practices made refills onerous and, coupled with high costs at private facilities, contributed to medication switching and discontinuation. All but three participants reported lacking resources and/or transportation to purchase ET. We also highlight how individual motivation, financial status, and social support helped some survivors overcome obstacles and remain adherent.

### Sample Characteristics

We analyzed 19 interviews with women who were of age 54 years on average (Table 2). Interviews typically lasted 45 minutes. All but three reported receiving radiotherapy. Approximately one third were 1-3 years from their mastectomy, whereas 68% were 3-5 years out from surgery. Sixty-eight percent received oncology care in Gaborone (South East); the rest were treated in Francistown (North East). However, women were from multiple districts across the country, mostly rural villages, which were 35-190 km away from the nearest public cancer hospital. Six women were living with HIV, and they tended to be younger.

**TABLE 2:** Sample Characteristics of BC Survivors

Characteristic	No. (%)
Female	19 (100)
Age, years, mean (SD)	54 (9.8)
Educational attainment	
Primary	7 (37)
Junior secondary	6 (31)
Senior secondary	3 (16)
Tertiary	3 (16)
Marital status	
Married	5 (26)
Single	11 (58)
Widowed	2 (11)
Missing	1 (5)
Employment status	
Full time	8 (42)
Part time	2 (11)
Not currently working	8 (42)
Missing	1 (5)
Home district	
Central	4 (21)
Kgatleng	1 (5)
Kweneng	4 (21)
North-East	2 (11)
North-West	1 (5)
South-East	3 (16)
Southern	4 (21)
Rural residence	15 (79)
HIV status	
HIV-positive	6 (32)
HIV-negative	13 (68)
BC stage	
I/II	8 (42)
III	11 (58)

Abbreviations: BC, breast cancer; SD, standard deviation.

### Limited ET Counseling

#### IHC Results Influenced Initiation

We identified three conflicting accounts of HR status from patient narratives and IHC results in medical records. For one woman whose IHC indicated her cancer was HR+, she explained, “according to the medical team, the type of cancer I have is not hormonal.” Although she should have been prescribed ET, she did not initiate due to the erroneous HR− result communication. Two with HR− results in their records reported taking ET “since treatment finished,” which may have been before IHC results were available. Although they “haven't missed any of my doses,” they did not recall the details of their results. Instead they mentioned, “when you come to the hospital and the doctor prescribes what can help, I have to accept it.”

The remaining results focus on 16 women who were correctly prescribed ET. All of these women reported initiating ET, and some described taking it for multiple years. Most women were prescribed tamoxifen (84%) or anastrozole (11%) and initiated during chemotherapy, radiation, or after treatment was completed.

#### Lack of ET Knowledge

Most women were aware that “these pills need to be taken for five years every month without fail,” yet they reported being confused about *why* they were taking ET (Table 3). Some women “didn't know anything,” including the medication name, so some referred to their prescription bottles to identify it. Most were unsure about how the medication helped them or what the benefits were explaining that they “take it for the sake of [taking] it.” Instead, they described wanting to follow doctors' instructions, suggesting patients with cancer “become vulnerable” and do not have a choice, so they agree to everything.

**TABLE 3:** Exemplary Quotes of Factors Contributing to ET Access and Adherence

Theme	Participant Quote
Initiation and limited counseling	
Lack of knowledge on ET purpose and side effects	“The health care workers said it can treat the disease […] I just took it without knowing it had side effects. They also did not explain to me - they only told me there is a pill I must take for 5 years.”—Stage III, 63 years
Side effects were reported but did not influence utilization	“I will take it for 5 years [… but] tamoxifen is troublesome: body aches, joint pains and hot flushes. The worst thing is forgetfulness.”—Stage III, 56 years
Adherence and challenges at public pharmacies	
Prescription policies and practices made refills difficult	“Going to Marina every month for pills [is difficult]. […] A doctor once wrote a prescription so that we can collect them from the clinic, but the pharmacy doesn't agree. […] The Pharmacy staff wrote a letter that I should bring to the clinic. After I brought it, the clinic was confused and kept on saying many things, in the end they said they are going to call…they later said the letter should be taken to another place. I ended up returning the letter because there was no proper procedure!”—Stage I, 72 years
Stockouts led to medication switching and discontinuation	“I had some breaks as it was not available. I managed to get it in February. I took it for one month and it is out of stock again […] I couldn't buy [privately] as I didn't have money.”—Stage III, 56 years
Women were highly motivated to obtain medication	“I hear it [tamoxifen] is sold at GPH and is cheap, about P70.00 for a month supply, so, I will go and hear what they are to say. At other outlets, it is very expensive.”—Stage III, 67 years
Access and high costs at private pharmacies	
Low SEP individuals discontinue	“If I don't have money, it becomes a problem. I stay alone and my kids have since moved out. I have to tell them in advance that I am running out of my pills. […] I haven't bought any. I just stayed without them. I once spent three months without them.”—Stage I, 72 years
Few women had financial resources or transportation to facilitate ET purchases	“Tamoxifen is out of stock […] it was not there. You can imagine for those who do not have money what will they do? For me, as I can buy for myself, it was not that difficult. Government should have everything that is required available so that an ordinary Motswana can access those. Also, they should expand. We cannot be getting treatment from Gaborone and Francistown only. Services should go to the people because others fail to get treatment. For example, someone may need to go to Francistown from Ghanzi, but they won't be able to travel because they won't have money. Treatment should be available across the places like for other diseases [HIV].”—Stage II, 47 years

Abbreviations: ET, endocrine therapy; GPH, Gaborone Private Hospital; SEP, socioeconomic position.

Only a few women recalled discussions with providers about the need for pills to prevent cancer from recurring and reiterated “my cancer is fueled by hormones.” Only two recalled receiving information about potential side effects of ET. One knew her medication “may change as time goes on” because “they change it for women who reach menopause.” Aside from these few women mentioning they received counseling about ET, we were unable to identify specific factors about the conversations or where women received information.

Provider knowledge about ET affected at least one survivor whose doctor erroneously advised her to stop ET for 6 weeks while receiving radiotherapy. However, another doctor “informed [her that she] should have never stopped taking it.”

#### Side Effects Did Not Influence Utilization

Some (25%) described tamoxifen as troublesome and reported experiencing body aches, joint pain, hot flashes, dizziness, and forgetfulness. However, most women did not link their symptoms to ET as they were largely unaware of the possible side effects. A few women were unsure if their symptoms were due to ET or chemotherapy: “I would vomit and experience some nausea. I did not know if it was [from] this pill or not.”

None of the women described stopping medication due to side effects. However, one participant reported severe leg pain while taking tamoxifen, so her provider switched her to anastrozole.

### Stockouts at Public Pharmacies Were Common

Hours-long wait times at public pharmacies were prohibitive as many women relied on others for transportation or were concerned about missing work. One participant recalled, “you arrive at 2:00 pm and leave at 5:00 pm. I don't have the patience.”

Over half of the sample recalled facilities being frequently out of stock of tamoxifen, sometimes for weeks or months. In one instance, the available pills expired and were no longer usable. When pharmacies were out of stock, few women had the financial resources to travel to and check commercial pharmacies.

Some refills were refused at private and clinic pharmacies due to policies/practices when women were told they did not have an acceptable prescription. For example, one woman was turned away after waiting in the queue because she forgot the refill prescription and the pharmacy had no record of her past prescription. Another tried to get medication from a clinic and was told to refill it elsewhere due to not being specifically written for that facility. Still, another expressed, “my main concern is that when the pills are out-of-stock, they [public providers] can't write a prescription for me to go and get them from the [private] pharmacy.”

Despite these challenges, nearly all participants were motivated to adhere and consistently sought refills. They were persistent in obtaining monthly prescriptions for refills, shopping around and locating affordable pills from private pharmacies that had medication in stock, and seeking or accepting financial and transportation assistance from family and community members.

### High Costs Associated With Private Pharmacies Exacerbated Barriers

#### Financial Barriers Were Common

When discussing public stockouts, many women echoed the sentiment “if you don't have money like I don't have money, it becomes difficult” to afford pills from private facilities. Many women described variation in prices between pharmacies as a major challenge to refill prescriptions. Some went multiple weeks without refills until the price was affordable. Lack of affordable transportation was also a barrier to accessing medication.

Medication discontinuation and switching were common when “the challenge is the medications are in short supply.” Two participants originally taking tamoxifen switched to anastrozole due to stockouts, explaining, “I went to the [private] chemist [who] told me they had a different one [anastrozole] […] I checked [a different] pharmacy yesterday and they still do not have.”

Some had resources to shop around when the public hospital was out of stock. Few could afford the costs at private pharmacies. These women acknowledged paying higher prices and traveling to other towns. In particular, one survivor “had to call my cousin to look for it in Kasane and send it here.” Financial assistance and other support from family/friends helped some pay for medication or other costs: “I sometimes receive a basket full of fruits from my friends, but sometimes I get money for transport.”

## DISCUSSION

This analysis of ET experiences among BC survivors found adherence was suboptimal due to health system barriers. We identified several themes women faced in initiating, accessing, and using ET: poor IHC result communication and counseling, frequent stockouts at public pharmacies, and high medication and transportation costs associated with private pharmacies. Side effects were reported but did not affect adherence. Although women were motivated to adhere, logistical barriers meant switching and discontinuation occurred.

A few women had conflicting IHC results and ET prescriptions, resulting in two HR− women taking tamoxifen for years and one HR+ woman never initiating ET. Although IHC is available in Botswana, there are frequent lapses in access due to supply of unstable reagents, faulty equipment, or laboratory personnel availability. Consequently, sometimes decisions regarding ET are made without HR results. Inconsistent or lack of IHC testing has also been an important barrier to guideline-concordant care in Tanzania,^57^ but in the Democratic Republic of the Congo, all patients with BC are prescribed tamoxifen due to unknown hormonal status.^58^

Most of our participants were unsure of ET benefits and side effects, which they attributed to limited provider counseling. Martei and colleagues identified similar inadequate information from providers affecting receipt of guideline-concordant cancer treatment in Botswana, although this did not include ET.^42^ Limited patient understanding throughout diagnosis and treatment is a well-documented barrier to care across SSA.^59-62^ It is possible that low ET knowledge among health care providers outside of specialized oncology centers may also contribute to lack of communication about the importance of adherence or alternative medications. Expanding oncology training to providers at primary clinics and hospitals^63^ and having nurses counsel and navigate may be feasible approaches for promoting ET adherence in low-resource settings.^32,64,65^

Although one quarter of our sample experienced side effects, no participants reported aches, pains, or hot flashes as a reason for discontinuing ET. Instead, they talked about chemotherapy and radiation side effects more generally, which others have also found can influence treatment noncompliance.^66,67^ In South Africa, younger patients with BC had worse ET adherence,^22^ with side effects being the main reason for discontinuation among WLWH.^25^ However, we and others did not observe differences in adherence among WLWH.^68^ Still comorbidities can predict adverse reactions to cancer drugs among patients with BC,^35^ so additional research on the combination of medications and motivations for adherence among women with comorbidities is needed.

Medication shortages, wait times, and prescription practice discrepancies between hospitals and pharmacies made it difficult for our participants to obtain ET. Multiple participants reported months-long discontinuation due to shortages. Delays in ET initiation and refills have also been reported in Ethiopia.^32^ Improving prescription processes, pharmacy capacity, and health infrastructure for medication procurement and distribution with cross-system electronic medical records may increase access to cancer care.^69^ Shortages also meant some women switched from tamoxifen to anastrozole, including young, likely premenopausal women, which is ineffective among women with intact ovarian function.^70,71^ Notably, in women with HR+ human epidermal growth factor receptor 2–negative disease with higher risk of recurrence, like many survivors in this study, current evidence recommends ovarian suppression and prolonged ET duration with or without CDK4/6 inhibitor therapy.^72,73^ Implementing such a change in SSA warrants future research as these intensive adjuvant hormonal therapies become standard of care in LMICs.

We also observed financial barriers to accessing ET, including medication and transportation costs. Patient SEP and distance to health facilities are well-documented barriers to treatment including ET in other African countries.^22,74,75^ For example, cancer medication pricing (including tamoxifen) was not affordable for patients paying out of pocket in Kenya, Uganda, and Rwanda.^29^ The financial toxicity of a BC diagnosis in SSA is clear: some patients and their households' SEP were negatively affected, which meant patients had to forgo care.^76^ Only a few households from our study had resources to purchase ET regularly. ET discontinuation has also been documented among Ethiopian patients reporting financial hardship and lack of transportation.^32^ Ensuring equitable access to BC treatment, including no/low cost of services, increases the likelihood of receiving treatment in SSA countries.^77^

These findings must be interpreted in context. Our sample included participants who received mastectomies; thus, we were unable to explore experiences of women who did not undergo surgery or who may have received breast-conserving surgery elsewhere. We did not explicitly assess menopausal status, which, along with age <50 years,^78^ predicts tamoxifen-related side effects.^79^ We also relied on self-reported adherence and were unable to confirm refills with pharmacy records. Despite these limitations, to our knowledge, this is the first qualitative description of experiences with and barriers to ET adherence among BC survivors in Botswana where most patients with BC are HR+.^38^

In conclusion, this study highlights areas for interventions to improve cancer care quality. Although we observed a few discrepancies with IHC result communication and ET initiation, most survivors with HR+ tumors initiated and tried to adhere when medication was in stock. Our findings suggest improvements are needed in counseling about ET importance as well as education and support for side effects. These data also emphasize that health system challenges must be addressed to ensure consistent access to affordable ET. Prioritizing ET accessibility remains critical to improve BC care quality and outcomes in Botswana and other LMICs.
